# Supplementary material for: Paclitaxel-Loaded, Pegylated Carboxylic Graphene Oxide with High Colloidal Stability, Sustained, pH-Responsive Release and Strong Anticancer Effects on Lung Cancer A549 Cell Line
Source: Pharmaceutics. 2024 Nov 14;16(11):1452. doi: 10.3390/pharmaceutics16111452 (PMC11597291; doi:10.3390/pharmaceutics16111452)
Supplement: Supplementary file 1 [file pharmaceutics-16-01452-s001.zip › pharmaceutics-3292312-supplementary.pdf]

Article

Paclitaxel-Loaded, Pegylated Carboxylic Graphene Oxide with High Colloidal Stability, Sustained, pH-Responsive Release and Strong Anticancer Effects on Lung Cancer A549 Cell Line

Athina Angelopoulou [1,*], Myria Papachristodoulou [1], Efstathia Voulgari [1], Andreas Mouikis [1], Panagiota Zygouri [2], Dimitrios P. Gournis [3,4] and Konstantinos Avgoustakis [1,*]

[1] Department of Pharmacy, Medical School, University of Patras, 26504 Patras, Greece; efivoulgari48@gmail.com (E.V.); andreas27997@gmail.com (A.M.)
[2] Department of Materials Science and Engineering, University of Ioannina, 45110 Ioannina, Greece;
pzygouri@gmail.com
[3] School of Chemical and Environmental Engineering, Technical University of Crete, 73100 Chania, Greece; dgournis@tuc.gr
[4] Institute of GeoEnergy, Foundation for Research and Technology-Hellas, 73100 Chania, Greece
* Correspondence: angelopoulou@upatras.gr (A.A.); avgoust@upatras.gr (K.A.); Tel.: +30-2610-962317 (K.A.)

Synthetic procedure of GO 2

Synthetic procedure of CGO 2

Characterization of GO and CGO 2

Figure S4. ^{1}H NMR Spectra 5

Figure S5. TGA of nCGO-PEG formulations 6

Figure S6. TEM of nCGO-PEG formulations 7

Figure S7. Stability of Main Peak and PDI 8

Figure S8. Toxicity of nCGO-PEG(10 kDa) at 5h 8

References 9

Synthetic procedure of GO: The oxidation of graphite was performed by adding 5 g of powdered graphite (purum, powder ≤ 0.2 mm, Fluka) to a mixture of concentrated sulfuric acid and nitric acid (200 mL, 95–97 wt.% and 100 mL, 65 wt.%, respectively) while cooling in an ice-water bath for 20 min. After that, 100 g of powdered $KClO_3$ (Fluka) was added to the mixture in small portions under vigorous stirring. The product was washed until the pH reached 6.0. Finally, the obtained sample was dried at room temperature.

Synthetic procedure of CGO: 500 mg of graphite oxide were dispersed in 250 mL of distilled water by stirring. 25 g of $ClCH_2COOH$ and 30 mL of NaOH (1M) were then added and the mixture was sonicated for 3 h. After that time an aqueous HCl solution (1M) was added in small portions until the pH value reached 7. The resulting mixture was centrifuged and washed with distilled water before being dried at room temperature.

Characterization of GO and CGO: XRD analysis was applied to justify the crystal structure of the synthesized GO and CGO (**Figure S1**). The XRD patterns of CGO are compared with those of GO and starting graphite. In the XRD pattern of the initial graphite (black line), we observe the characteristic diffraction peak at 2θ = 26.46°, which is distinctive to graphite. The basal spacing of CGO, as deduced from the reflection at 2θ = 10.7°, is 8.23 Å [1], while for GO, the d_{001} reflection at 2θ = 12.7° is calculated at 7.0 Å. This difference in the interlayer space between two adjacent layers of GO and CGO is due to the increased number of carboxyl groups after the carboxylation process.

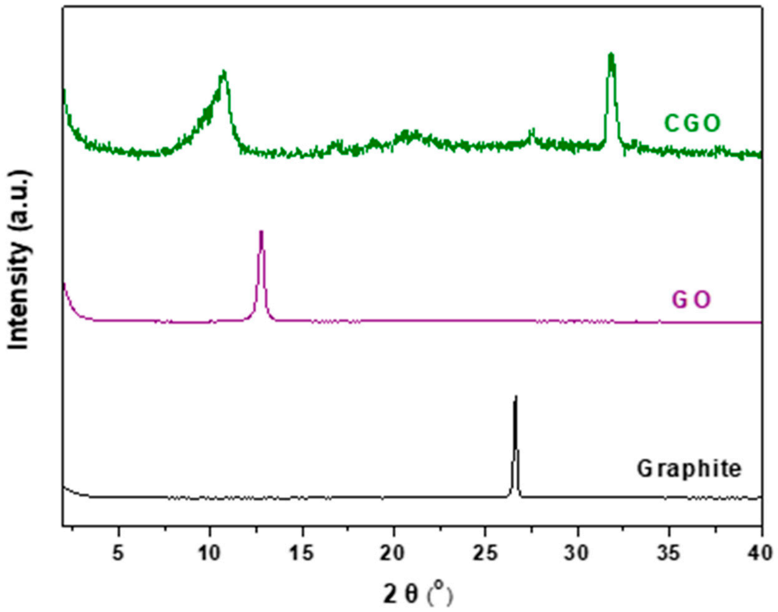

Figure S1. XRD pattern of graphite (black line), GO (purple line) and CGO (green line).

In **Figure S2**, FT-IR spectra were performed to investigate the functional groups GO and CGO. In the FT-IR spectrum of graphite (black line), there is no characteristic peak because of no asymmetric stretching vibrations. As shown in the FT-IR spectrum of GO (purple line), the bands centered at 3420, 1710, 1630, 1395 cm^{-1}, and 1060 cm^{-1} are attributed to the stretching vibrations of –OH, C=O, C=C, C-O, and C–O-(epoxy) groups, respectively [2, 3]. Concerning the FT-IR spectrum of CGO (green line), the characteristic

peaks of GO appear in the spectrum of CGO as well. However, the peak at 1710 cm^{-1} is more intense due to the increase of carboxyl groups after modification of GO. Moreover, the band of GO at 1630 cm^{-1} has been downshifted to 1602 $cm^{-1,}$ and this is an indication of the increase of carboxylic groups because they have high electronegativity attracting electrons from the graphitic domain, leaving low charge density on C=C bonds [4].

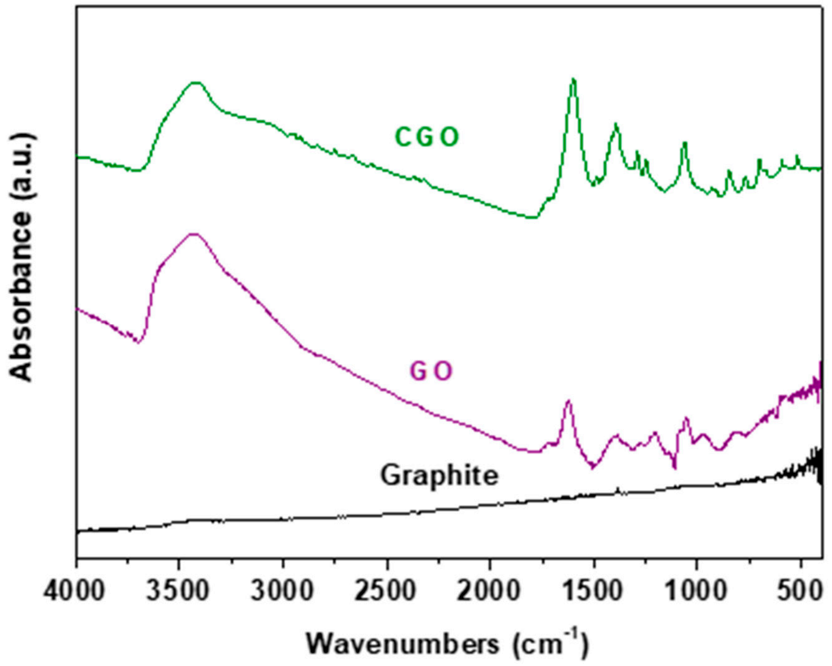

Figure S2. FTIR spectra of graphite (black line), GO (purple line) and CGO (green line).

The Raman spectra of initial graphite (black line), GO (purple line), and carboxylated GO (green light) are presented in **Figure S3**. The two major peaks at ~1346 cm^{-1} (D band) and ~1596 cm^{-1} (G band) for GO and carboxylated GO are due to the existence of sp^3 hybridized carbon atoms mentioned as defects for the graphitic domain (D band) and caused due to the oxidation process which causes the brake of C=C bonds on the aromatic rings creating oxygen functionalities. G band is characteristic of the sp^2 hybridized carbon atoms due to the stretching vibration of C-C bonds of graphene. The ratio of the D to G band is a bit higher for CGO ID/IG=0.95 compared to GO ID/IG=0.88, declaring more defects on the final carboxylated structure. However, the difference in the ratios is so tiny that it shows that the carboxylation took place mainly on the oxygen functionalities after the oxidation of graphite and not in the planar structure of graphene. As for initial graphite, the ratio of D to G band is very low because of the negligible defects of the graphitic plane. Moreover, the ratio between 2D and D+G bands (I_{2D}/I_{D+G}) expresses the destruction of sp^2 carbons and is lower for CGO, which implies that the oxidation occurred more efficiently due to the increased number of carboxyl groups [5].

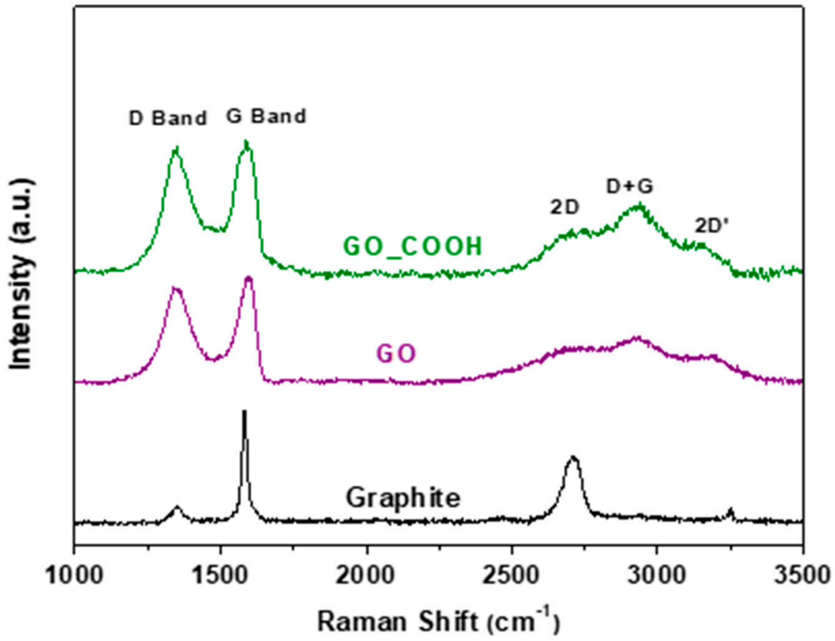

Figure S3. Raman spectra of graphite (black line), GO (purple line) and CGO (green line).

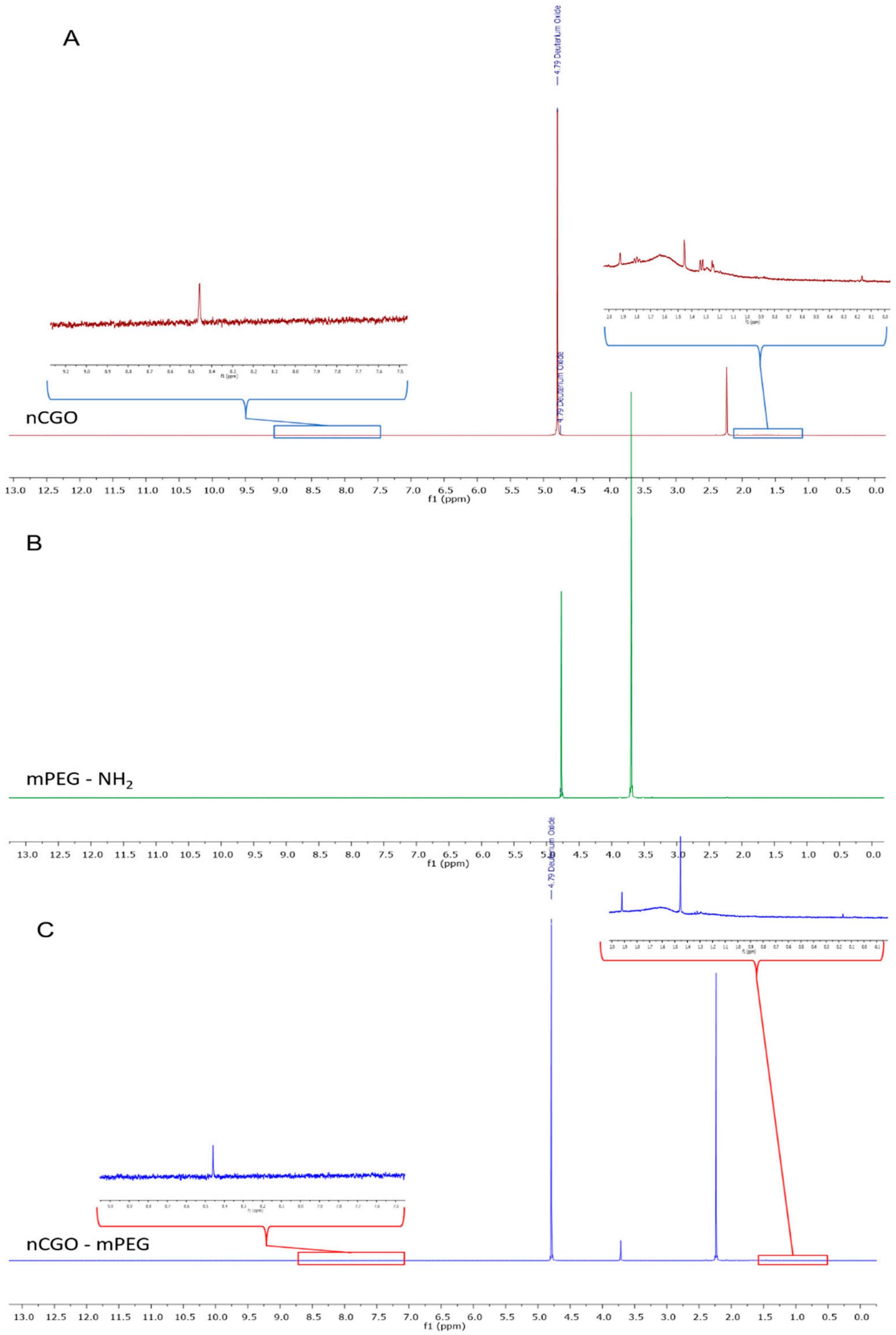

Figure S4. ^{1}H NMR Spectra of A) nCGO formulations, B) PEG (10 kDa) -NH_2 polymer, C) nCGO-PEG(10 kDa) formulations in D_2O. Comparatively of the nCGO-PEG (C) spectra revealed a shift at 2.21 ppm attributed to the nCGO methylene (–CH_2-) protons, varied weak peaks at the range 1.0 - 2.0 ppm of the aliphatic area attributed to the - CH - CH protons of GO, a peak at 3.69 ppm ascribed to the methylene (-CH_2-) protons of the PEG units and a peak at 8.5 ppm attributed to the aromatic protons of the graphite lattice

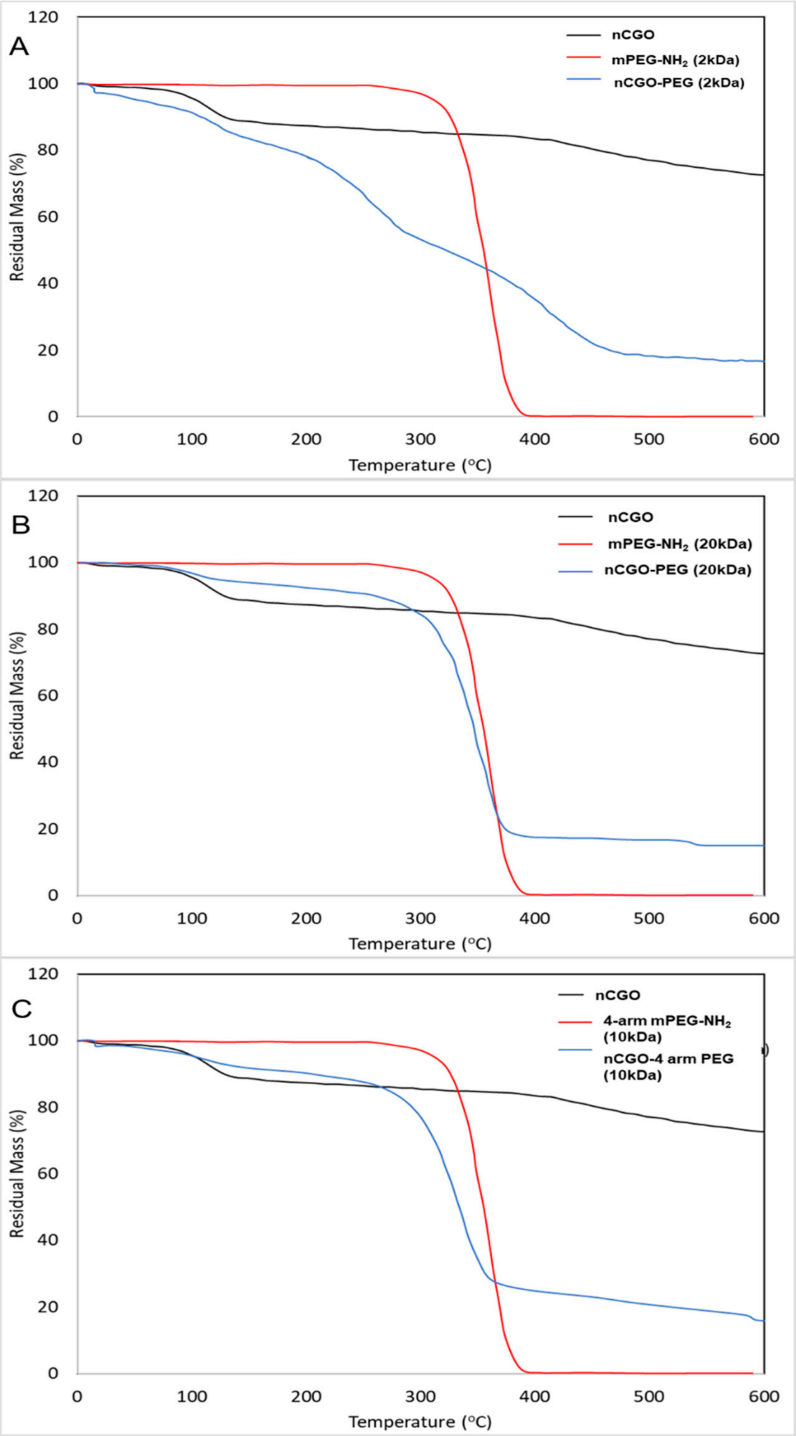

Figure S5. TGA of nCGO-PEG formulations (blue line) in comparison with the specific mPEG-NH_2 polymer (red line) and nCGO (black line). The thermograms are of A) nCGO-PEG(2 kDa), B) nCGO-PEG(20 kDa) and C) nCGO-4-arm PEG(10 kDa).

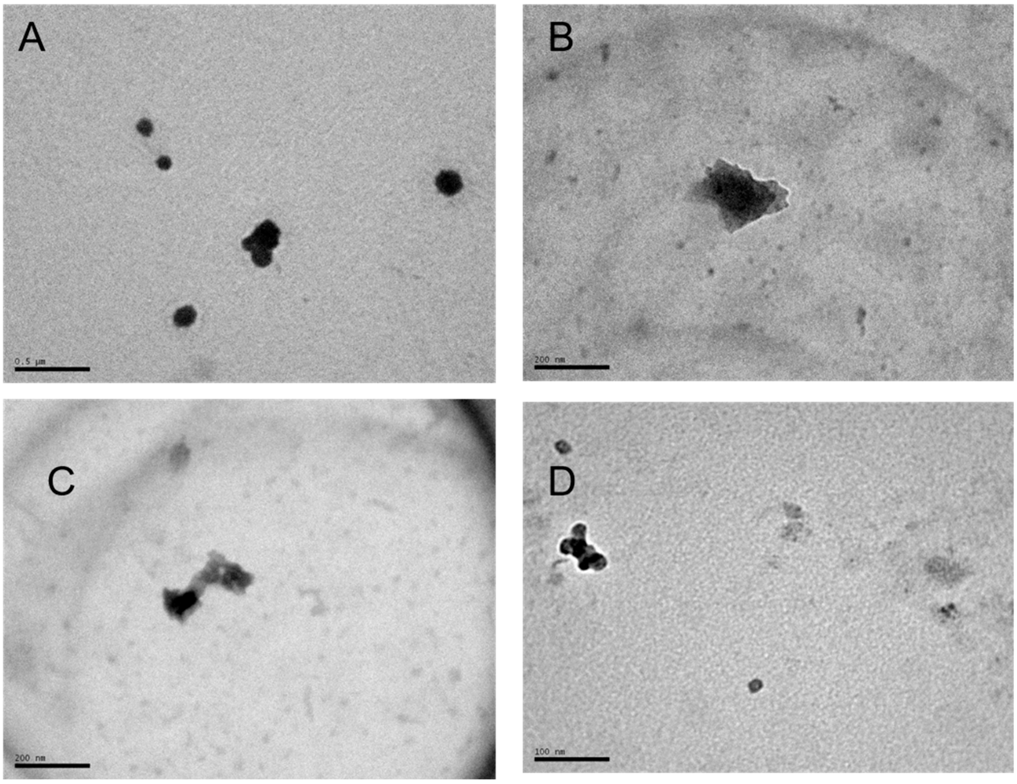

Figure S6. TEM micrographs of nCGO (A), nCGO-PEG(2 kDa) (B), nCGO-PEG(20 kDa) (C), and nCGO-4 arm PEG(10 kDa). The scale bar in all micrographs is 200 nm.

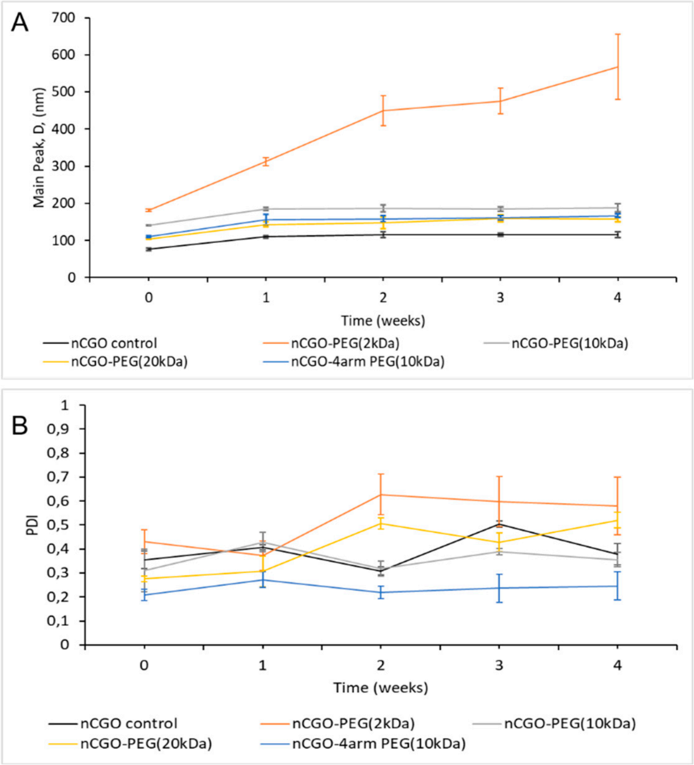

Figure S7. Colloidal stability of nCGO and nCGO-PEG formulations in biological media (RPMI 1640, and PBS). Average variations of A) main peak by DLS and B) PDI

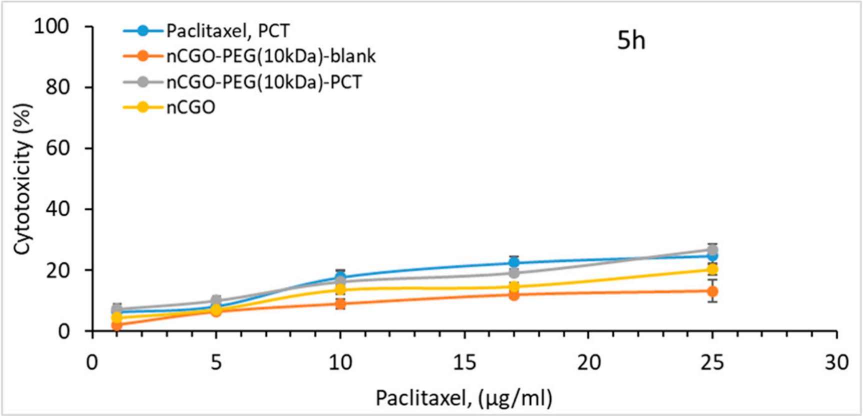

Figure S8. Cytotoxicity of nCGO-PEG(10 kDa) against A549 cancer cells at 5 hours (h) of incubation

References

[1]. Zygouri, P.; Spyrou, K.; Papayannis, D.K.; Asimakopoulos, G.; Dounousi, E.; Stamatis, H.; Gournis, D.; Rudolf, P. Comparative Study of Various Graphene Oxide Structures as Efficient Drug Release Systems for Ibuprofen. *AppliedChem 2(2)* ***2022***, *93-105*. https://doi.org/10.3390/appliedchem2020006

[2]. Travlou, N.A.; Kyzas, G.Z.; Lazaridis, N.K.; Deliyanni, E.A. Functionalization of Graphite Oxide with Magnetic Chitosan for the Preparation of a Nanocomposite Dye Adsorbent. *Langmuir 29(5)* ***2013***, *1657-1668*. https://doi.org/10.1021/la304696y

[3]. Jin, Z.; Wang, X.; Sun, Y.; Ai, Y.; Wang, X. Adsorption of 4-n-Nonylphenol and Bisphenol-A on Magnetic Reduced Graphene Oxides: A Combined Experimental and Theoretical Studies. *Environmental Science & Technology 49(15)* ***2015***, *9168-9175*. https://doi.org/10.1021/acs.est.5b02022

[4]. Chen, Y.; Xie, B.; Ren, Y.; Yu, M.; Qu, Y.; Xie, T.; Zhang, Y.; Wu, Y. Designed nitrogen doping of few-layer graphene functionalized by selective oxygenic groups. *Nanoscale Research Letters 9(1)* ***2014***, *646*. https://doi.org/10.1186/1556-276X-9-646

[5]. Zhan, D.; Ni, Z. ; Chen, W.; Sun, L.; Luo, Z.; Lai, L.; Yu, T.; Wee, A.T.S.; Shen, Z. Electronic structure of graphite oxide and thermally reduced graphite oxide. *Carbon 49(4)* ***2011***, *1362-1366*. https://doi.org/10.1016/j.carbon.2010.12.002
